# The Role of Gluten in Celiac Disease and Type 1 Diabetes

**DOI:** 10.3390/nu7095329

**Published:** 2015-08-26

**Authors:** Gloria Serena, Stephanie Camhi, Craig Sturgeon, Shu Yan, Alessio Fasano

**Affiliations:** 1 Center for Celiac Research, Mucosal Immunology and Biology Research Center, Massachusetts General Hospital and Division of Pediatric Gastroenterology and Nutrition, Massachusetts General Hospital for Children, Boston, MA 02114, USA; E-Mails: gserena@mgh.harvard.edu (G.S.); sscamhi@partners.org (S.C.); csturgeon@mgh.harvard.edu (C.S.); syan4@mgh.harvard.edu (S.Y.); 2 Graduate Program in Life Sciences, University of Maryland School of Medicine, Baltimore, MD 21201, USA

**Keywords:** celiac disease, type 1 diabetes, gluten

## Abstract

Celiac disease (CD) and type 1 diabetes (T1D) are autoimmune conditions in which dietary gluten has been proven or suggested to play a pathogenic role. In CD; gluten is established as the instigator of autoimmunity; the autoimmune process is halted by removing gluten from the diet; which allows for resolution of celiac autoimmune enteropathy and subsequent normalization of serological markers of the disease. However; an analogous causative agent has not yet been identified for T1D. Nevertheless; the role of dietary gluten in development of T1D and the potentially beneficial effect of removing gluten from the diet of patients with T1D are still debated. In this review; we discuss the comorbid occurrence of CD and T1D and explore current evidences for the specific role of gluten in both conditions; specifically focusing on current evidence on the effect of gluten on the immune system and the gut microbiota.

## 1. Celiac Disease

Celiac disease (CD) is an autoimmune enteropathy caused by the ingestion of gluten in genetically susceptible individuals. It is characterized by the presence of autoimmune antibodies, systemic clinical manifestations, small intestinal enteropathy and genetic predisposition [1]. CD is a T-cell mediated disorder where gliadin derived peptides activate immune cells in the gut lamina propria and recruit infiltrating T lymphocytes, which initiate an adaptive Th1 response and concurrent increase of interferon gamma (IFN-γ) and interleukin-15 (IL-15). This leads to the activation of intraepithelial lymphocyte toxicity which results in profound tissue remodeling.

The etiology of CD is influenced by both environmental and genetic factors. The most characterized genetic contribution to CD is the human leukocyte antigen system (HLA), contributing to 40% of genetic variance [2]. Major histocompatibility coplex (MHC) class II HLA DQ2 and DQ8 confer the greatest disease susceptibility. The majority of patients carry variants of DQ2 (95%) encoded by alleles DQA1*05/DQB*02 and a minority (5%) carries DQ8 encoded by DQA1*03/DQB1*03:02 alleles. In addition, there is evidence for gene dosage effect with increased risk for those with homozygous allotypes [3]. Genome-wide association studies and a recent dense fine mapping study report 57 non HLA loci, which account for 18% of the genetic variance [4]. Of note, HLA and other genetic susceptibility are necessary but not sufficient for disease development. Additionally, environmental causative agents for CD, other than gliadin, have been explored.

Several studies have shown an association between active CD and gastrointestinal dysbiosis characterized by higher amount of *Proteobacteria* and *Bacteroidetes* and associated with a reduced abundance of the phylum *Firmicutes* during the acute phase of the disease [5]. A proof of concept study from our group revealed that genetically predisposed infants present a specific fecal microbiota in which *Bacteroides* are reduced and *Firmicutes* are more abundant before the onset of the disease [6]. Together, these data suggest a possible causative role of dysbiosis in the onset of CD.

Prevalence of CD is between 1% and 2% of the total population in North America, South America, the Middle East and North Africa, and there is preliminary evidence for similar rates in Asian populations [7]. The disease incidence is also increased in first degree family members of those with CD (10%–15%) and in individuals with other autoimmune diseases [8]. Of the affected population, studies suggest that only 10%–15% is actually diagnosed. Despite high estimates of undiagnosed cases, recent studies have also indicated a surprisingly sharp increase in the prevalence of CD over the past decades, with North America and Europe experiencing the highest increase [9].

Classical presentation of CD consists mainly of gastrointestinal symptoms associated with malabsorption including diarrhea, steatorrhea, weight loss, or failure to thrive. Other extra-intestinal symptoms include iron deficiency, recurrent abdominal pain, aphthous stomatitis, chronic fatigue, short stature and reduced bone density [10]. Serum testing is extremely reliable and forms the first line of testing for CD. Patients are first screened for serum IgA anti-tissue transglutaminse antibodies if they are not IgA deficient [11]. In individuals with IgA deficiency, serum IgG anti-tissue transglutamminase antibody levels are measured instead of traditional markers. Recently, IgG anti-deamidated gliadin has emerged as an alternative test, due to its better sensitivity and specificity (capability of discriminating false positives and false negatives) [12]. A more specific, but more expensive and operator dependent, test is sometimes used to confirm borderline results: anti endomysium IgA. While performing the serologic screening, patients should remain on a gluten containing diet in order to maintain high sensitivity of test results. Finally, a confirmed diagnosis requires a small intestinal biopsy. Histological changes should show increased number of intraepithelial lymphocytes, elongated crypts and at least partial villous atrophy [10].

The definitive treatment for CD is complete elimination of the offending gluten. A gluten free diet (GFD) should cause no side effects, since gluten has limited nutritional value, but consumption of certain nutrients, in particular fibers, iron, calcium and folates tends to be lower in the GFD [12]. Strict compliance is necessary when adhering to a GFD as even small amounts of contamination can prove problematic [9]. Maximum contamination has recently been defined as 20 ppm by both the Codex Alimentarius and Food and Drug Administration [13]. Intestinal healing and decrease of serologic markers should begin between 6 and 24 months following the initiation of GFD [12]. Currently, the only available treatment for CD patients is a strict GFD. This therapy, however, fails to induce complete improvement in 7%–30% of patients. Thus, the number of studies evaluating alternative therapeutic strategies for CD, such as genetically modified gluten, inhibitors of zonulin—the regulator of intestinal tight junctions—or supplementary probiotics, have recently increased [14,15,16,17].

## 2. Celiac Disease and Gluten

Gluten is a complex molecule contained in several grains such as wheat, rye and barley [12,18]. The major proteic components that characterize gluten are glutenin polymers and gliadin monomers. Glutenins can be subdivided into low and high molecular weight proteins, while the gliadin protein family contains α-, β-, γ- and ω- types [19]. Both glutenins and gliadins are characterized by a high amount of prolines (20%) and glutamines (40%) that protect them from complete degradation in the gastrointestinal tract and make them difficult to digest [20].

The link between onset of CD and ingestion of gluten containing grains was established around 1950. Since then, the role of gliadin as the environmental factor for CD has been well established [12,18]. Several studies revealed the capability of gliadin peptides to trigger an immune response in CD patients. Castellanos-Rubio and colleagues showed that intestinal biopsies from active CD patients stimulated *in vitro* with gliadin express a vast range of pro-inflammatory cytokines derived from a Th1/Th17 driven adaptive immune response [21]. Furthermore, Palova *et al.* reported that peripheral blood mononuclear cells (PBMC) from CD patients responded to gliadin by secreting interleukin 1 beta (IL-1β) and interleukin-18 (IL-18) [22]. Recently, IL-15 has also been found to be up-regulated in the epithelium and the lamina propria of patients with active CD [23,24].

**Figure 1:**
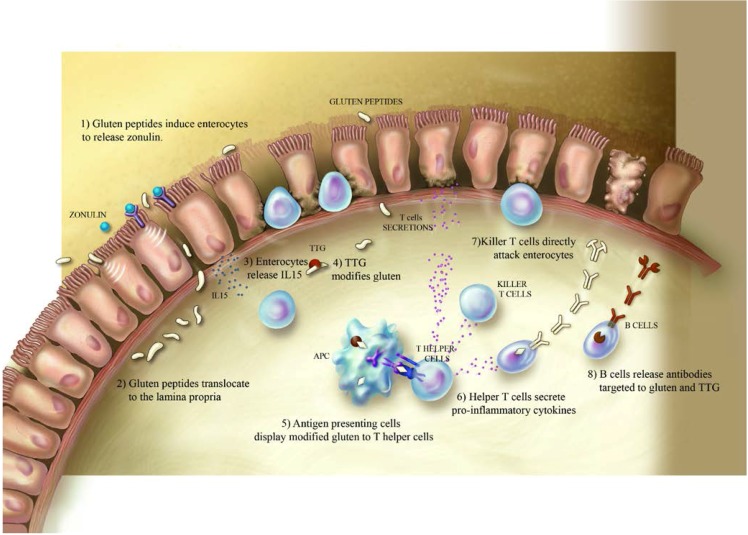
Mechanisms by which ingested gluten triggers celiac disease: digested gluten interacts with epithelial cells in the small intestine and triggers the disruption of tight junctions (1). The consequent increased intestinal permeability leads to the translocation of gluten peptides to the lamina propria (2) where they induce the production of IL-15 (3). In the lamina propria, gluten peptides are modified by tissue transglutaminase enzymes (4) and trigger an adaptive immune response (5–8).

Presently, the chain of events by which gliadin triggers onset of clinical disease is hypothesized as the following: after oral ingestion, partially digested gliadin peptides interact with the small intestinal mucosa and trigger an innate immune response characterized by release of IL-8 and IL-15 from epithelial cells and lamina propria dendritic cells [25,26]. IL-8 is a potent chemo-attractant and its production leads to the immediate recruitment of neutrophils in the lamina propria, while the release of IL-15 induces enterocyte apopotosis via NKG2D^+^ cells. Specific gliadin peptides interact with CXCR3 receptors expressed on the epithetlium’s apical side [27]. This interaction triggers the release of zonulin leading to increase antigen trafficking [28,29]. Gliadin peptides are then translocated into the lamina propria where they are deamidated by transglutaminase 2 [30]. Consequently to the deamidation, the peptides interact with macrophages and dendritic cells of the intestinal submucosa [31]. The following Th1/Th17 driven adaptive immune response is characterized by high production of pro-inflmmatory cytokines IFN-γ, tumor necrosis factor-α (TNF-α) and interleukin-17 (IL-17), which further increase intestinal permeability and provoke damage in the intestinal mucosa [32] (Figure 1).

Several studies have focused on the specific components of gluten that play a role in the various steps of disease onset. Many epitopes have been described and associated respectively with increased gut permeability, cytotoxic or immunomodulatory activities [33]. The majority of these epitopes are found in α- and ω-gliadins. The 57-89 peptide (33-mer) α-gliadin fragment has been particularly well studied. It contains most of the epitopes and has been reported to have immunomodulatory effects [34]. Shan *et al.* identified three distinct T cell epitopes within its aminoacidic sequence and they demonstrated its strong proteolitic resistance during digestion. Other known peptides are the cytotoxic peptide, the gut permeating peptides and the IL-8 releasing peptide [35].

As with many other autoimmune diseases, CD has been characterized by an increased worldwide prevalence in recent decades. Rubio-Tapia *et al.* showed that the incidence of CD in the USA has increased five-fold in fifty years [36]; similar results have been reported in Finland, where the overall prevalence of CD increased from 1.05% to 1.99% in 20 years [37]. Catassi *et al.* confirmed the increase in incidence of the disease in the past three decades and demonstrated that the loss of immunological tolerance to gluten can also occur later in adulthood [9].

The reasons behind the increased prevalence of CD in the last 50 years are not fully understood. The rapid change in incidence suggests that this higher prevalence cannot be attributed exclusively to genetic changes in the populations, but rather to environmental factors. The hygiene hypothesis has long been considered as the most plausible explanation for the increased incidence of autoimmune diseases; however, data from developing countries suggest that this may not be the case for CD. Several studies have considered variables that may be related to the increased incidence of CD, focusing mostly on timing of gluten introduction, amount of gluten supplemented to the diet, and the effects of breast feeding.

The introduction of gluten to Man’s diet has been reported to occur around 10,000 years ago; since then, its consumption has expanded up to five-fold [38]. Many hypothesize that the increased gluten intake of the last few decades paired with the introduction of a westernized diet in many countries may play a critical role in the increased prevalence of CD worldwide. In the last fifty years, an increase in gluten consumption has been reported in countries like Italy and Sweden. The corresponding increase in prevalence of CD in the same countries suggests that the amount of gluten consumed may be considered a major risk factor for development of CD [39,40]. This hypothesis appears to be corroborated by studies that show the introduction of a small amount of gluten between four and six months of age reduces the risk of the disease [41]. A retrospective analysis study explored whether the increased incidence of CD might be due to an increased gluten content in wheat derived from wheat breeding. Domesticated modern wheat has been shown to significantly differ from wild wheat: modern wheat has larger grains and higher protein content [38]. Interestingly, the author of this study did not find clear evidence to corroborate this hypothesis. Furthermore, the lack of sufficient data regarding the incidence of CD per year made it difficult to determine if the pro capita intake of wheat or gluten might have played a role in the incidence of CD. Another important aspect speculated to be a risk factor for CD is the time of gluten introduction in the infant diet. In the mid-1980s, the Swedish epidemic of symptomatic CD coincided with the new dietary recommendation of not feeding gluten-containing foods to infants until six months of age. Interestingly, the incidence of CD decreased when an earlier introduction of gluten (>4 months) was reintroduced as the standard of care in Sweden [40]. Conversely, two recent studies that followed infants deemed as high risk of CD, due to first degree relation to a CD patient, concluded that the timing of gluten introduction was not a significant factor in determining disease onset, although a later introduction of gluten was shown to be associated with a delayed onset of the disease [3,42]. The same studies also considered the effects of additional biological and environmental factors such as breast feeding and genetic predisposition to CD. Particularly during the first months of life, breast-feeding is well-regarded as the optimal feeding mode for infants. The advantages of breast-feeding in the health of infants have been reported by several studies [43,44]. Human milk aids in growth and supports the child’s neural and immunological development. However, the question whether breast-feeding can protect against the development of CD has been a matter of discussion for long time. An analysis of the Swedish CD epidemic reported that the development of CD in children under two years of age was reduced if they were still breast-fed when dietary gluten was introduced and that this “protective effect” of breast-feeding was even more pronounced if the infants were breast-fed also after the introduction of gluten [45]. Lionetti *et al.* showed that breast feeding did not modify the risk of CD among at risk-infants [3]. Similar data were confirmed by another study in which the authors concluded that CD development is not influenced by the duration of breast-feeding or by continuing breast-feeding after introduction of gluten [46].

Several studies have focused on relating intestinal dysbiosis to various diseases, among which CD is included. It has been shown that the dysbiosis characterizing active CD patients is partially reversible and linked to the presence of gluten in the diet. Nadal *et al.* found that the initiation of a GFD normalized the increase of gram-negative bacteria, *Bacteroides* and *E. coli* groups that characterized children with active CD [47]. Similarly, a study from Collado *et al.* reported that the elimination of gluten from the diet of pediatric patients led to a reduction in *E. coli* and *Staphylococcus*, which were found to be in higher abundance during the active state of the disease [5]. The beneficial effects of a GFD on the microflora and its metabolic function seem to be related to diet duration [48].

## 3. Type-1 Diabetes

T1D is an autoimmune disorder caused by the destruction of the insulin producing β-cells of the pancreas [49]. Although it is widely accepted that adults can also develop T1D, the highest incidence rate is found in adolescents [50,51,52]. Epidemiological studies have estimated the worldwide prevalence of T1D to be less than 1%. Recent evidences, however, suggest that its incidence has been increasing 3% per year [53]. Some large scale studies, such as The Environmental Determinants of Diabetes in the Young (TEDDY) [54], The Diabetes AutoImmunity Study in the Young (DAISY) [55], and TrialNet [56], have been initiated to identify potential environmental triggers and biomarkers for T1D so that, in the future, intervention may be possible to delay or even prevent the development of this condition.

The disease’s first manifestations develop when lack of insulin prevents cells from adequate glucose uptake, which is necessary and vital to cell function. Classic symptoms include polyuria, polydipsia, weight loss, fatigue, and hyperglycemia, which, if left untreated, can lead to a coma and ultimately to death [57]. Diagnosis of diabetes includes fasting blood glucose higher than 126 mg/dL, any blood glucose of 200 mg/dL or an abnormal oral glucose-tolerance test [58]. Since 2009, the American Diabetes Association has modified the guidelines for diabetes diagnosis to include the measurement of glycated hemoglobin levels (A1C). This reflects the amount of blood glucose attached to hemoglobin and it is considered positive if higher than 6.5% on two occasions.

An important serological component that characterizes T1D and distinguishes it from type 2 diabetes is the presence of auto-antibodies against β-cell auto-antigens. Islet cell antibodies (ICA) were the first auto-antibodies described to be associated with the development of T1D [59]. In addition to ICA, more than 90% of T1D patients have auto-antibodies to insulin (IAA) [60], glutamic acid decarboxylase (GADA) [61], and protein tyrosine phosphatase like protein (IA2). These auto-antibodies are also used to identify subjects at high risk of developing the disease, since they are present months to years before symptom onset and can be detected in serum as early as six months of age in genetically susceptible individuals [62].

The exact pathogenesis of T1D is not completely understood, however it is well accepted that both genetic and environmental factors play a role. The genetic locus with the highest association to T1D is the HLA locus, which accounts for about 50% of the genetic load [63]. HLA DQ2 and DQ8 loci are the strongest determinants of diabetes susceptibility and HLA DR4 and DR3 have been shown to be associated with T1D. The heterozygous DR3/DR4 genotype is associated with the highest risk of disease onset, followed by DR3/DR3 and DR4/DR4 homozygosity [64].

Other genes that have also been associated with T1D are IL-2 receptor α [65], cytotoxic T lymphocyte antigen (CTLA4) [66], protein tyrosine phosphatase non-receptor 22 (PTPN22) [67], intercellular adhesion molecule 1 (ICAM1) [68], and the insulin gene (INS) [69]. In addition to genetic components, it is suggested that environmental factors may play a key role in T1D onset. The incidence of diabetes is increasing faster than can be explained by genetics alone, which is likely due to environmental changes.

Although many possible contributing environmental factors have been identified, to date none have been confirmed as a clear causative agent of T1D. The most frequently proposed candidates are viruses, such as enteroviruses [70,71,72,73], rotavirus [74,75], and rubella [52]. The potential role of these pathogens in the onset of T1D would corroborate the hygiene hypothesis [76]. In the last decades, changes in microbiome composition has also been suggested to be involved in the development of T1D by either altering intestinal permeability or modifying immune system regulation [77]. Studies in non-obese diabetic (NOD) mice in specific pathogen-free or germ free conditions confirmed the role of the microbiome in the regulation of islet specific autoimmunity [78]. Human studies have shown a correlation between T1D onset and a lower diversity and stability of the intestinal microflora, including a decreased ratio between *Firmicutes* and *Bacteroidetes* [79,80]. Other environmental factors such as climate and nutrition have been shown to be risk factors for T1D, but also in these cases there is no strong evidence that these agents are causative of T1D [81].

## 4. Type 1 Diabetes and Gluten

Although numerous studies have suggested a potentially pathogenic role of gluten in T1D, the exact mechanisms by which it may play a role in the onset and development of T1D are not yet fully understood (Box 1).

Studies in human samples have reported that upon stimulation with wheat proteins or their components, patients with T1D showed a heightened proliferative T cell response as compared to control patients [82,83,84]. PBMC from these patients produced significantly more pro-inflammatory cytokines compared to control subjects. Similarly, Klemetti *et al.* found that PBMC from T1D patients proliferated more as compared to controls when stimulated with wheat proteins [85]. This proliferative behavior, however, appeared to depend on the duration of disease. While 24% of patients with newly diagnosed T1D responded to the stimulus, only 15% of patients with longer duration of T1D and 5% of non-disease control subjects showed proliferative behavior. Such observed alterations characterizing the adaptive immune response are not restricted to the periphery. Jejunal biopsies from patients with T1D have increased CD25^+^ mononuclear cell density as compared to control patients when stimulated with gliadin [83]. Furthermore, observations *in vivo* showed that rectal administration of gluten in T1D patients induced infiltration of CD3 and Tγδ rectal lymphocytes in a subgroup of patients [84]. Dietary gluten has also been shown to affect components of the innate immune system, such as dendritic cells [86].

The use of animal models, such as the NOD mice or BBdp rats, have allowed for a better understanding of the effect of dietary gluten on T1D progression, even as early as during gestation. The cumulative incidence of T1D has been shown to be reduced in offspring of NOD mice fed a GFD during pregnancy [87]. Similarly, it has been found that diabetes onset is delayed in offspring of NOD mice fed a modified diet (in which wheat barley proteins were absent) during gestation [88]. These results do not seem to translate to humans, as Lamb *et al.* found the maternal frequency of ingestion of gluten-containing foods during the final trimester of pregnancy to have no effect on the development of T1D in offspring [89].

A reduced incidence of diabetes was observed in NOD mouse offspring from mothers fed a basal (gluten-containing) diet during gestation but a wheat barley protein-free (WBP) diet at or after weaning [88]. Similarly, offspring from basal fed mothers who started a WBP free diet after weaning had reduced T1D incidence compared to offspring with identical gestational feeding practices but who received a basal diet after weaning. Taken together, these findings suggest that the infant diet during weaning is a stronger modulator of T1D development than maternal feeding practices during gestation. In humans, the effect of timing of gluten introduction on T1D and/or IA development remains controversial. Some studies report a marked increase in risk of IA development and/or T1D incidence in infants introduced to gluten prior to three months of age or later than six months of age [90,91,92,93], while others report no effect whatsoever [94,95]. Interestingly, a Swedish group has reported that the introduction of gluten after six months of age increased risk of GADA or IA-2A positivity almost six-fold in children who also were introduced to cow’s milk formula before two months of age [93]. This finding therefore suggests a synergistic effect of early exposure to cow’s milk formula and late exposure to gluten.

There is strong evidence that removal of gluten from the diet can selectively be protective against development of diabetes [96,97]. Incidence of hypoglycemia is higher in NOD mice maintained on a gluten-containing diet, while a GFD casein-based diet serves to reduce incidence of hypoglycemia, delay onset of T1D, and reduce IA titers [98]. Additionally, incidence of diabetes in NOD mice maintained on a WBP-free diet is reduced as compared to mice maintained on a standard gluten containing diet (51% *versus* 81%), and IA titers are lower in NOD mice maintained on a lifelong WBP diet or a WBP diet following weaning [88]. Hypothesized mechanisms for these advantageous effects of GFD include modification of intestinal permeability and the composition of the gut microbiota. Watts *et al.* found an increase in intraluminal zonulin in BBdp rats just prior to hypoglycemia, which correlated with increased intestinal permeability in the animals [99].

The elimination of gluten from the diet has been shown to be advantageous also by modulating the microbiota composition. Marietta and colleagues observed that NOD mice fed a standard gluten-containing diet had increased *Barnesiella*, *Bifidobacterium*, *Tannerella* and *Turcibacter* microbes, as compared to an increase in *Akkermansia* and *Bacteroides* microbes in GFD NOD mice [96]. In addition, NOD mice fed a GFD were observed to have greater total microbial richness than NOD mice fed a gluten-containing diet. Hansen and colleagues also reported distinct microbial signatures associated with diet and found a marked increase in the bacterial phylum *Verrucomicrobia*, *TM7* and *Proteobacteria* in GFD NOD mice [87]. In BBdp animal models, total islet number was increased in rats housed in germ-free *versus* specific pathogen-free (SPF) conditions [98]. In these SPF conditions, beta cell mass was lowest in BBdp animals fed a gluten-containing diet. Taken together, these findings suggest that microbes play an important role in modulating islet and beta cell integrity, which in turn modulates development of T1D.

Box 1 **Main findings about the correlation between gluten and the onset of T1D.**

- *In vitro* studies:
  - Upon stimulation with wheat proteins T cells from T1D patients show a higher proliferative and pro-inflammatory response than T cells from control subjects [85].
  - Upon stimulation with gliadin, jejunal biopsies from T1D patients show increased CD25+ cell density as compared to control patients [83].
- *In vivo* studies:
  - Incidence of T1D is reduced in offspring of NOD mice fed a GFD during pregnancy [87].
  - Removal of gluten from the diet selectively protects NOD mice from developing T1D [96].
  - FGD casein-based diet reduces incidence of hypoglycemia, delays onset of T1D and reduces IA titers in NOD mice [98].
  - Gluten containing diet alters the composition of the innate immune system in BALB/c and NOD mice and it is correlated with an increased expression of dendritic cells activation markers in NOD mice [86].
- *Human studies:*
  - Introduction of gluten in the diet of infants prior to 3 months of age or later than 6 months is correlated with an increase in risk of IA development and T1D incidence [91,92].
  - Introduction of gluten after 6 months of age increases risk of GADA and IA-2A positivity [93].

Box 1 summarizes the main findings about the correlation between gluten and the onset of T1D.

## 5. Comorbidity between Celiac Disease and Type-1 Diabetes

The association between CD and T1D was first reported in the late 1960s. The prevalence of CD in patients with T1D is estimated to fall between 1.4% and 19.7% [100,101,102,103]. This comorbidity can largely be attributed to overlapping genetic HLA risk loci; in both conditions, the HLA-DQ2 and DQ8 genes have been shown to be important determinants of disease susceptibility. Additionally, some non-HLA genes such as PTPN22 and CTLA4 have been associated with either CD or T1D [67].

Usually, T1D develops prior to diagnosis of CD, though cases exist in which CD develops as the primary disease and T1D develops later in life [104,105,106,107]. In the latter case, individuals are significantly older at T1D onset than those who develop it prior to CD. Additionally, individuals who develop CD and T1D tend to be younger at diabetes onset than those with T1D who never go on to develop CD [108].

Because of the high rate of comorbidity between CD and T1D, there is great need for a more efficient, targeted diagnostic approach. Numerous findings suggest that many CD cases in T1D patients would be overlooked by use of a single serological screening at T1D onset. Tsouka and colleagues recently found that 12.2% of patients who eventually came to have dual diagnosis of CD and T1D first presented with at least one negative celiac screen (in serum) after T1D diagnosis [101]. On average, 47.8 months lapsed between the first negative serological test and a positive result suggesting CD autoimmunity (CDA). Additionally, a cohort by Bakker and colleagues found that 42% of T1D patients who came to develop CD were not diagnosed (with CD) until 10 years following T1D onset [106]. It is also important to recognize that serological markers of CD in patients with T1D may not always prove specific or reliable. It has been recently reported that a small subset of T1D children found to have CDA will spontaneously normalize their celiac serology with a median time of 1.3 years and thus will not be diagnosed with overt CD [102,109]. Though the authors did not classify their participants in this way, those found to have transiently elevated or fluctuating levels of anti-tTG IgA are generally termed as having potential CD. In the work of Castellaneta and colleagues, a small group of T1D patients with potential CD were found to have only an infiltrative lesion or entirely normal mucosa upon inspection with upper endoscopy.

The importance of a systematically correct and timely diagnosis is highlighted by the fact that additional autoimmune diseases are often reported to occur secondary to onset of comorbid T1D and CD with autoimmune thyroid disease (ATD) presenting most commonly [105,106]. Furthermore, Shah *et al.* found that children with dual diagnosis are three times more likely to be vitamin D deficient as compared to healthy children without autoimmune conditions [110]. In the cohort, risk of vitamin D deficiency for children with T1D alone was increased over healthy children by only 1.5 times. This raises the question of whether bone health is affected for children diagnosed with both T1D and CD. Joshi and colleagues reported that children with T1D found to have CDA have lower bone mineral density at the whole body and lumbar spine compared to children with T1D alone. In terms of other medical complications that may arise due to dual diagnosis of CD and T1D, findings are mixed regarding whether individuals with dual diagnosis are at greater risk of retinopathy or nephropathy compared to those with T1D alone (Table 1) [111].

It has been suggested that symptoms associated with CD are more difficult to control in patients that have also T1D. Mackinder *et al.* showed that levels of tTG (IgA) took longer to normalize for children with dual diagnosis compared to children diagnosed with CD alone [100]. Another group found the majority of T1D children diagnosed with concomitant CD presented with gastrointestinal symptoms, all of which resolved with adoption of the gluten free diet. In addition to this, BMI and weight SDS improved significantly in these children, and a trend was observed towards an increase in insulin requirement only for children who were compliant with the gluten free diet [107]. However, it remains to be elucidated whether metabolic control improves for T1D patients with underlying concomitant CD after adoption of the GFD. Two different studies reported higher HbA1c levels and more frequent hypoglycemic episodes in T1D children found to have CDA as compared to children with T1D alone [107,111]. Conversely, others have reported no difference in insulin requirement or growth status between dual diagnosis individuals and individuals with T1D alone [106].

A growing body of evidence suggests that the beneficial effects of a GFD (for patients with concomitant CD) may actually protect against development of further T1D-related complications. Compared to T1D patients without CD, Bakker and colleagues found a lower prevalence of retinopathy and lower total cholesterol in adult T1D patients with concomitant CD. Warncke *et al.*, similarly, reported lower absolute systolic blood pressure in T1D patients with CD as compared to those without CD, suggesting that this modulatory effect is attributed to the GFD [108]. In the same cohort, patients with dual diagnosis had significantly lower levels of HDL cholesterol as compared to individuals with T1D alone; after institution of a GFD, these levels increased significantly and the difference between groups disappeared. It is possible that these improvements in cholesterol level could be due to the normalization of the intestinal mucosa with adoption of a GFD, demonstrating a beneficial effect of adhering to the diet.

**Table 1:** Main features of celiac disease and type 1 diabetes.

Feature	Celiac Disease	Type 1 Diabetes
Wordwide incidence	0.6% *–1%	<1%
Contribution of HLA genes	HLA DQ2 (DQA1*05-DQB1*02) HLA DQ8 (DQA1*03-DQB1*03:02)	HLADQ2 and/or DQ8 (DRB1*0401-DQB1*03:02 and DRB1*0301-DQB1*0201)
Non-HLA candidate genes	CTLA4, PTPN22,CD28, ICOS, MYO9B	CTLA4, PTPN22, MIC-A
Symptoms	Diarrhea, steatorrhea, weight loss, failure to thrive, iron deficiency, abdominal pain, reduced bone density, chronic fatigue, growth failure.	Polyuria, polydipsia, extensive hunger, weight loss, chronic fatigue, reduced bone density, growth failure, hyperglycemia.
Diagnosis	Small intestinal biopsy, generally with supporting serological testing.Serologic tests: IgA anti-tTG, IgG anti-tTG, IgA anti-EMA, IgG DGP.	Blood test: Fasting blood glucose level, oral glucose tolerance test, A1C. Serologic tests: ICA, IAA, GADA, IA2 antibodies
Comorbidities	Type 1 diabetes, Down syndrome, Turner syndrome, William’s syndrome, vitiligo, Addison’s disease, hyperparathyroidism, neuropathy, IgA nephropathy, psoriasis.	Celiac disease, Grave’s disease, Hashimoto’s disease, Addison disease, vitiligo, autoimmune thyroid disease.
Pathogenesis	Enteropathy is due to dysregulation of the innate and adaptive immune system. Alteration of intestinal permeability.	Autoimmune destruction of pancreatic insulin-producing β-cells by an adaptive and innate immune response. Alteration of intestinal permeability.

## 6. Conclusions

In this review, we focused on the role of gluten as an important player in the pathogenesis of CD and T1D. The high rate of comorbidity between these two autoimmune diseases and their rapidly increasing prevalence in the last few decades underscore the importance of screening in high risk patients and the need to further explore and detail the contributory role of environmental factors that may be involved.
